# Standard basic emergency obstetric and neonatal care training in Addis Ababa; trainees reaction and knowledge acquisition

**DOI:** 10.1186/1472-6920-14-201

**Published:** 2014-09-24

**Authors:** Alemnesh H Mirkuzie, Mitike Molla Sisay, Mulu Muleta Bedane

**Affiliations:** Center for International Health, University of Bergen, Årstadv 21, Overlegedanielsenshus, Bergen 5020 Norway; School of Public Health, College of Health Sciences, Tikur Anbessa Hospital, Second Floor, Room Number 58, Post Box 40860, Addis Ababa, Ethiopia; WAHA International, University of Gondar, Post box 41822, Gondar, Ethiopia

**Keywords:** Addis Ababa, BEmONC, Competency, In-service, Knowledge based mastery, Providers, Training, Training evaluation

## Abstract

**Background:**

In 2010, the Federal Ministry of Health of Ethiopia (FMOH) has developed standard Basic Emergency Obstetric and Neonatal Care (BEmONC) in-service training curricula to respond to the high demand for competency in EmONC. However, the effectiveness of the training curricula has not been well documented. A collaborative intervention project in Addis Ababa has trained providers using the standard BEmONC curricula where this paper presents Krikpartick level 1 and level 2 evaluation of the training.

**Methods:**

The project has been conducted in 10 randomly selected public health centers (HC) in Addis Ababa. Providers working in the labour wards of the selected HCs have received the standard BEmONC training between May and July 2013. Using standard tools, trainees’ reaction to the course and factual knowledge during the immediate post-course and six months after the training were assessed. Descriptive statistics and t-tests were done.

**Results:**

Of the total 82 providers who received the training, 30 (36.6%) were male, 61 (74.4%) were midwives. Providers’ work experiences ranged from 1 month to 37 years. Seventy-four (89%) providers reported that the training was appropriate for their work, 95% reported that the training have updated their knowledge & skills, while 27 (32.9%) reported that the training facilities & arrangements were unsatisfactory. The mean immediate post-course knowledge score was 83.5% and 33 (40%) providers did not achieve knowledge-based mastery in their first attempt. The midwives were more likely to achieve knowledge-based mastery than the nurses (p < 0.05). The mean knowledge score six-months post-training was 80.2% and 40% have scored knowledge based mastery.

**Conclusions:**

Being one of the first papers reporting the implementation of the standard in-service BEmONC training curriculum, we have identified an important limitation on the course evaluations of the curriculum, which need urgent consideration. The majority of the trainees has reported favourable reaction to the training, but many of them did not achieve knowledge-based mastery in the immediate post training although the knowledge retention six months post training was encouraging.

## Background

In recent years, Ethiopia shows appreciable progresses in maternal and child health and already achieved MDG4 and is on track to achieve MDG5 [1]. These achievements are largely attributed to successes in community based interventions and interventions targeting the post-neonatal period [2]. Despite progresses made, the proportions of women giving birth in health facility are still below the threshold to ensure improved maternal and neonatal outcomes. In MDG countdown reports it is highlighted that Emergency Obstetric and Neonatal Care (EmONC) should be a priority intervention to enable the country to further advance in maternal and neonatal health as it promotes the provision of life saving obstetric and neonatal care procedures [2–5]. For years, Ethiopia has been striving to ensure the availability and accessibility of quality EmONC services across the country since it was first introduced in the country in 1998 as part of the “Saving the Mothers” project in few selected sites [6, 7].

Nevertheless, 10 years after its introduction a nationwide facility based EmONC assessment survey that was conducted in 2008 revealed gaps in availability, accessibility and quality of EmONC services across the country. Informed by the findings, efforts have been made to scale up the EmONC services and to avail the necessary inputs for delivering quality service across the country. Two years later, another nationwide hospital based EmONC survey in 2010 has shown advances in personnel, supplies, logistics and supervision, but not in providers competency [8]. To bridge the competency gaps affecting the quality of EmONC, the government has directed its focus on standardizing the EmONC training curricula and its implementation. In 2010, the Federal Ministry of Health of Ethiopia (FMOH) in collaboration with local and international partners developed a competency based standard BEmONC training curricula for in-service training of midwives and nurses [9].

The training uses a mastery learning approach, where trainees are required to achieve mastery in knowledge and skills on obstetric and neonatal care topics covered in the training. Moreover, Krikpatrick’s 4 level training evaluation is employed to assess training effectiveness [10]. Level 1 is the evaluation of trainees’ reaction to the training and this assessment would inform about the acceptability of the training as a favorable reaction to the course might yield better learning and training outcomes. It also helps to assess areas that need emphasis in the training. Level 2 is evaluation of learning of knowledge and skills, level 3 is evaluation of change of behaviour following the training and level 4 is an evaluation of outcomes in terms of improved care and reduced mortality and morbidity (Table 1).

**Table 1:** **The four level Krikpatrick’s evaluation of training effectiveness**

Levels		Descriptions
**Level 1**	Reaction	To what degree trainees react favourably to the training?
**Level 2**	Learning	To what degree trainees acquiring the intended knowledge, skills and attitudes?
**Level 3**	Behaviour	To what degree trainees apply what they learned during the training when they are back to on the job
**Level 4**	Outcomes	To what degree targeted outcomes occur as a result of training and subsequent reinforcement

To contribute to the efforts in improving maternal and newborn health outcomes, a collaborative project between the Centre for International Health, University of Bergen, the Addis Ababa City Council Health Bureau and the Addis Ababa University, Ethiopia has been conducted in Addis Ababa. The project intends to improve the quality of basic EmONC in Addis Ababa city and to ensure equitable access through intensive hands on skills training using simulation technology with MamaNatalie and NeoNatalie. In the project the standard BEmONC training materials were used to train midwives and nurses working in 10 randomly selected public health centers (HCs) between May and July 2013. This paper is one of the series of papers from the project, which present findings from a longitudinal design that measured knowledge-based mastery during the immediate post-course and six months after the training and also reports trainees’ reaction to the training.

## Methods

Addis Ababa, the capital of Ethiopia is administratively divided into 10 sub-cities. Of the over 3.5 million people living in the city, about 70% are mothers and children. Over 70 public HCs and five public hospitals under the Addis Ababa City Administration Health Bureau are offering maternal and child health care to the majority of the city dwellers. All the public HCs are primary care units and are the first contact point in the continuum of maternal and newborn health care. These HCs are also providing BEmONC, while all the public hospitals provide comprehensive EmONC. A referral network has established from the public HCs to the hospitals for mothers and babies who are requiring advanced interventions.

This study was part of the collaborative intervention project in Addis Ababa. The project has got ethical approval from the Addis Ababa City Administration Health Bureau Ethics Committee and by the regional Ethics committee in West Norway. Study permits were obtained from the Addis Ababa City Administration Health Burea, the health bureaus of the 10 sub-cities and from the project HCs. The main objective of the project is to improve the quality of basic EmONC in Addis Ababa and to ensure equitable access. For improving quality, intensive hands on skills training using low cost and low tech simulators (MamNatalie and NeoNatalie) were provided. For bridging the equity gaps, the project targeted public HC where the majority of poor and disadvantaged women are accessing EmONC services. Of the 24 HC providing delivery care services in the city, ten HC one from each sub city was randomly selected for the project.

**Figure 1:**
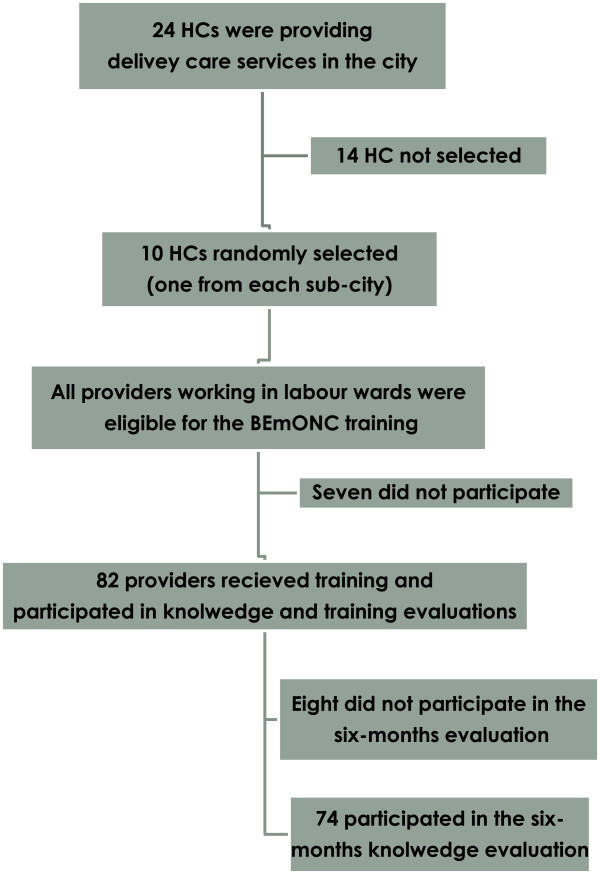
**Study flow chart.**

There were 89 midwives and nurses who were working in the delivery wards of the project HCs. Of these eligible providers, seven did not receive the training (One for social reason while the rest had already received the training recently). Eighty two providers received the standard BEmONC training in four rounds where each round of the training took three weeks in accordance with the standard set by the FMOH [9] (Figure 1). The Ethiopian Midwifery Association having vast experiences in implementing the standard BEmONC training across the country conducted the training under close supervision of the project leader and project team members.

The first eight days of the training were classroom theoretical sessions complemented with demonstration, videos, case studies and role plays. The last ten days were for the skills training using demonstrations and clinical sessions until trainees achieve mastery in selected skills with checklist based evaluations. Prior to the start of the course, pre-course assessment was done to identify knowledge gaps among trainees on the management of shock, bleeding before and after birth, normal labour, third stage of labour, pre-eclampsia/eclampsia, partograph use, puerperal sepsis and on neonatal resuscitation.

By the end of the theoretical sessions, there was an immediate post-course evaluation to assess knowledge-based mastery using a tool that contained 36 multiple-choice questions on nine major topics; 1) infection prevention practices, 2) vaginal bleeding in early pregnancy, 3) rapid initial assessment and management of shock, 4) childbirth care, 5) unsatisfactory progress of labour, 6) mal-presentation and malposition, 6) headache, blurred vision, convulsions or loss of consciousness, elevated blood pressure, 7) vaginal bleeding after child birth, 8) fever and 9) new born care. Trainees who answered 31 questions correctly out of the 36 or those who scored ≥85% were considered to have achieved knowledge-based mastery according to the standard BEmONC training curriculum [9]. Trainees who failed to achieve knowledge-based mastery in the first attempt sat for re-exam until they recorded mastery. However, this paper presents the knowledge-based mastery recorded in their first attempt. Six-month post training 74 (90.2%) providers participated in a knowledge retention evaluation (Figure 1). All the providers were approached in their respective workplace to fill out the same tool that was used to assess the knowledge-based mastery during the immediate post-course. The aim of this evaluation was to assess knowledge retention six months after the training.

By the end of the third week of the training, trainees were asked to evaluate the course in eight major areas in a scale ranging from strongly disagree to strongly agree. Assessed topics included 1) For the work I do, the training was appropriate 2) Training facilities & arrangements were satisfactory 3) The facilitators were knowledgeable & skilled 4) The facilitators were fair and friendly 5) Training objectives were met 6) The training was updating my knowledge & skills 7) Teaching aids were useful and 8) Practice in the clinical areas was important & helpful. The findings are presented in percentages, mean, standard deviation and student t-tests.

## Results

Eight two providers had received the standard BEmONC training in the project. The providers were between the age of 20 and 59 with mean of age 28.1 year. Thirty (36.6%) of the trainees were female and 61 (74.4%) were midwife by profession. Years of professional experience ranged from 1 month to 37 years whereby over 75% had less than seven years of work experiences (Table 2).

**Table 2:** **Characteristics of providers who received standard BEmONC training in Addis Ababa between May 6, and July 7, 2013**

Variable	Number (%)
**Age**	
20-24	30 (36.6)
25-29	32 (39.0)
≥30	20 (24.4)
**Sex**	
Male	30 (36.6)
**Profession**	
Midwife	61 (74.4)
Nurse	21 (25.6)
**Years of experience**	
0 - 1 year	23 (28.0)
1.1-3.7 years	18 (22.0)
3.8 -7 years	22 (26.8)
7.1 - 37 years	19 (23.2)
**Previous in-service BEmONC training**	
Yes	4 (4.9)

The pre-course evaluation revealed knowledge gaps in eight of the nine topics assessed. Major knowledge gaps were seen on “bleeding during pregnancy and labor”, “partograph use” and “fever during and after childbirth”. The lowest mean knowledge score was recorded on “partogoraph use”. All the trainees correctly answered the question regarding “newborn resuscitation” (Table 3). There were no significant differences in the pre-course knowledge score by age, sex, profession and years of work experience (not shown).

**Table 3:** **Pre-course knowledge scores among the 82 providers who received standard BEmONC training between May 6 and July 7, 2013**

Topics assessed	Mean score (number of question)	Standard deviation
Management of shock; rapid initial assessment	2.33 (out of 3)	0.58
Bleeding during pregnancy and labor	2 00 (out of 4)	0.94
Bleeding after childbirth	1.76 (out of 3)	0.62
Management of third stage of labor	2.23 (out of 3)	0.83
Headaches, blurred vision, convulsions, loss of consciousness or elevated blood pressure	2.76 (out of 4)	0.88
Partograph use	0.48 (out of 1)	0.51
Normal labor and childbirth	6.30 (out of 9)	1.35
Fever during and after childbirth	1.09 (out of 2)	0.54
Newborn resuscitation	1.00 (out of 1)	0.00

### Krikpatrick level 2: learning

**Figure 2:**
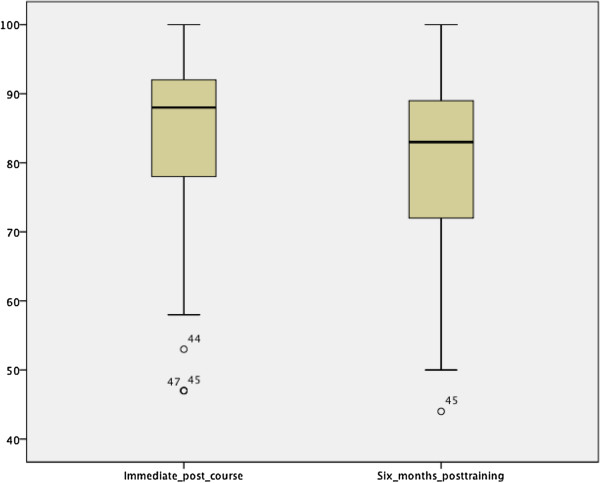
**Immediate post-course and six-months post training knowledge scores of providers who received standard BEmONC training between May 6 and July 7 2013.**

The immediate post-course mean knowledge score was 83.5% (SD 13.5) with a minimum of 47% and a maximum of 100%. Forty percent (33) failed to record knowledge-based mastery (a score of ≥85) in their first attempt (Figure 2). In a bivariate regression analyses, nurses were 4.4 times more likely to fail the post-course assessment compared to midwives (OR 4.4 95% CI 1.5, 12.7). Female course participants were over 3 times more likely to fail the post-course knowledge assessment compared to their male counterparts (Odds ratio 3.3 95% CI 1.2, 9.0). No significant association was observed between failing post-course assessment and years of work experiences. In a multivariate logistic regression model, being a midwife and being a male participant were independent predictors for scoring a passing grade in the post-course.

### Knowledge retention

Seventy-four (90.2%) of the trainees had participated in the evaluation of factual knowledge retention six months post training. The six months post training knowledge score ranged between 44% and 100% with a mean score of 80.2% (SD 12.2) and 40% recorded knowledge-based mastery. In a bivariate regression analyses, the midwives were 3.6 times more likely to record knowledge-based mastery (score ≥85%) than the nurses (OR 3.6 95% CI 1.2, 11.2).

The six-months post training score was not significantly associated with years of work experiences (OR 1.3 95% CI 0.9, 1.9) and sex (OR1.8, 95% CI 0.7, 4.6). In a multivariate logistic regression model controlling for sex, years of professional experience, age and previous in-service EmOC training; profession was an independent predictor for recording knowledge-based mastery six-months post training. Midwives were over 3 times more likely to score a passing point than nurses (OR 3.5 95% CI 1.1,11.1).

To examine the degree of knowledge decay over time, the immediate post-course mean knowledge score was compared with that of the six-months post training score using paired sample t-test. The result showed no significant decay in providers’ knowledge over the six-month period following the training with the mean difference of 2.9% (95% CI 0.6, 5.7).

### Krikpatrick level 1: trainees’ evaluation of the course

By the end of the third week trainees were asked to do an overall training evaluation. In the evaluation, 74 (89.0%) thought that the training was appropriate for the work they do and 76 out of 80 (95.0%) thought that the training has updated their knowledge & skills. For 27 (32.9%) of the trainees the training facilities & arrangements were found unsatisfactory (Table 4).

**Table 4:** **Trainees evaluation of the standard BEmONC training that was conducted between May 6 and July 7, 2013**

	Strongly agree	Agree	Undecided	Disagree	Strongly disagree
For the work I do, the training was appropriate	60	13	9		
Training facilities & arrangements were satisfactory	37	18	27		
The facilitators were knowledgeable & skilled	55	17	5		
The facilitators were fair and friendly	63	17	2		
Training objectives were met	63	19			
The Training were updating my knowledge & skills	54	22	4		
Teaching aids were useful	57	23	2		
Practice in the clinical areas was important & helpful	54	21	3	1	2

## Discussion

This paper presents Krikpatrick level 1 and level 2 evaluation of the standard BEmONC training conducted in Addis Ababa. In Krikptrick level 1 evaluations, all the providers reacted positively to the training and unanimously agreed on the relevance of the training for their work. In Krikpatrick level 2, during the immediate post course 40% of the providers did not achieve knowledge-based mastery with insignificant factual knowledge decay six months post training. The nurses generally recorded lower knowledge-based mastery during the immediate post training and in the six months post training compared to the midwives.

In Krikpatrick level 1 training evaluation, trainee reaction to the training showed that, trainees were content with the training; on the appropriateness of the course for their work, trainers’ competence, interpersonal communication with the trainers, with the training materials, training objectives and on the skills training. This highlights the relevance and acceptability of the standard BEmONC training. In a study by Ameh from Somaliland, where similar training curricula were used, trainees had reacted positively to the emergency obstetric care training [11]. However, in our study about one third of the trainees were not satisfied with the training facilities, as the training halls were not spacious enough. We have conducted the training in Menellik II Midwifery College; one of the best setup in Addis Ababa in terms of availing the necessary training materials and equipment. The training venues were however small that could have a negative effect on the learning of some trainees. Future training programs should take into consideration the need for a spacious training venue for BEmONC training.

The pre-course knowledge assessment revealed that trainees had marginal knowledge on identifying and managing most obstetric emergencies suggesting the need for in-service knowledge updating. Less than half of the trainees reported having sufficient knowledge on partograph use. Consistent with our finding other studies in Addis Ababa identified providers’ poor competence in using partograph for monitoring labour, although the majority reported that they are routinely using it [12, 13]. Harvey and his colleagues have made similar observations in two African and three Latin American countries where knowledge about partograph use was found to be low [14]. Partograph when properly filled out and interpreted, can assist providers to make correct judgment to handle obstetric complications, whereas inappropriate use of it might increase referrals and undesirable maternal and neonatal outcomes [15].

The major weakness we have identified in the implementation of the standard BEmONC training curricula is on the training evaluation, in particular on knowledge evaluations. Urgent considerations are called for the curriculum to objectively document knowledge change before and after training. Following the eight days classroom theoretical sessions, 40% of the trainees have failed to achieve knowledge-based mastery. These would challenge the effectiveness of the training in terms of meeting its intended objectives, although we could not able to compare with the pre-test knowledge score. Several studies have shown significant changes in knowledge on obstetric and neonatal care following pre-service and in-service trainings in resource poor settings, although making direct comparisons among the different studies are difficult [10, 11, 16–18]. A randomized controlled trial from the United Kingdom that employed a 1-2 days training have shown significant improvement in knowledge pertinent to emergency obstetric management [19].

Compared to the nurses, midwives were more likely to achieve knowledge-based mastery during the immediate post-course and in the six months post-training. This finding is not surprising as there are obvious differences in the level of training and level of exposure to obstetric and neonatal conditions between the nurses and the midwives. Although, nurses are one group of skilled attendants, the pre-service nursing curricula have limited coverage of courses to ensure midwifery competencies. A study by Fullerton and colleagues who conducted a study in three African countries, including Ethiopia shows that the nursing curricula do not conform to the standard set by the International Confederation of Midwives in terms of course contents and level of practical exposure [20]. Secondly, increasingly, nurses in Addis Ababa are having limited in-service exposure to emergency obstetric and neonatal conditions as well as limited opportunities to practice what they have acquired from pre-service and in-service trainings as they have taken over by the midwives. Similarly, a study from the UK shows that senior doctors who are exposed to emergency obstetric conditions record higher pre-test score of emergency obstetric knowledge than midwives who are mostly involved in attending normal labour, highlighting the values of clinical exposures and experiences for ensuring competency [19].

The level of factual knowledge decay six months following the training was insignificant. Consistent with our findings, studies from Ethiopia and in Liberia among community health cadres who received training on community maternal and newborn health care have shown a high degree of knowledge retention at 18 to12 months after the training respectively [17, 18]. Moreover, the Ethiopian study documented that actionable topics (topics that require to be applied) are more likely to be retained [18]. Croft and colleagues who have documented outcomes of training for midwives and doctors in intrapartum emergencies also report sustained knowledge retention six and 12 months post training [21].

Reporting only Krikpatrick level 1 and level 2 training evaluation is one of the limitations of this paper. One cannot be sure that improvement in knowledge could lead to improved work performance and improved overall maternal and neonatal health outcomes. However, when the project data collection is over, we are intending to report Krikpatrick level 3 and 4 evaluations. The other limitation of the study is that, different tools were used for assessing pre-course and post-course knowledge in accordance with the standard BEmONC curriculum, hence, we were not able to measure knowledge gained from the training.

## Conclusions

Standardizing the BEmONC training curricula is an important step to respond to the high demand for competency in EmONC in Ethiopia. Being one of the first papers reporting the implementation of the standard in-service BEmONC training curriculum, we have identified an important limitation on the course evaluations, which needs urgent consideration. The majority of the trainees has reported favourable reaction to the training, but many of them did not achieve knowledge-based mastery in the immediate post training although the knowledge retention six months post training was encouraging. Tailored training approach might be important to attain optimal training outcomes and impacts for the different category of professionals having various background knowledge and skills.
